# Adhesion of ***Escherichia coli*** under flow conditions reveals potential novel effects of FimH mutations

**DOI:** 10.1007/s10096-016-2820-8

**Published:** 2016-11-05

**Authors:** T. Feenstra, M. S. Thøgersen, E. Wieser, A. Peschel, M. J. Ball, R. Brandes, S. C. Satchell, T. Stockner, F. M. Aarestrup, A. J. Rees, R. Kain

**Affiliations:** 1 Clinical Institute of Pathology, Medical University of Vienna, Währinger Gürtel 18–20, 1090 Vienna, Austria; 2 National Food Institute, Research Group for Genomic Epidemiology, Technical University of Denmark, Søltofts Plads 221, 2800 Kongens Lyngby, Denmark; 3 Department of Biotechnology and Biomedicine, Bacterial Ecophysiology and Biotechnology Group, Technical University of Denmark, Matematiktorvet 301, 2800 Kongens Lyngby, Denmark; 4 Department of Nephrology, Ipswich Hospital, Heath Road, Ipswich, IP4 5PD UK; 5 Academic Renal Unit, University of Bristol, Southmead Hospital, Bristol, UK; 6 Institute of Pharmacology, Center for Physiology and Pharmacology, Medical University of Vienna, Währingerstrasse 13A, 1090 Vienna, Austria

## Abstract

FimH-mediated adhesion of *Escherichia coli* to bladder epithelium is a prerequisite for urinary tract infections. FimH is also essential for blood-borne bacterial dissemination, but the mechanisms are poorly understood. The purpose of this study was to assess the influence of different FimH mutations on bacterial adhesion using a novel adhesion assay, which models the physiological flow conditions bacteria are exposed to. We introduced 12 different point mutations in the mannose binding pocket of FimH in an *E. coli* strain expressing type 1 fimbriae only (MSC95-FimH). We compared the bacterial adhesion of each mutant across several commonly used adhesion assays, including agglutination of yeast, adhesion to mono- and tri-mannosylated substrates, and static adhesion to bladder epithelial and endothelial cells. We performed a comparison of these assays to a novel method that we developed to study bacterial adhesion to mammalian cells under flow conditions. We showed that *E. coli* MSC95-FimH adheres more efficiently to microvascular endothelium than to bladder epithelium, and that only endothelium supports adhesion at physiological shear stress. The results confirmed that mannose binding pocket mutations abrogated adhesion. We demonstrated that FimH residues E50 and T53 are crucial for adhesion under flow conditions. The coating of endothelial cells on biochips and modelling of physiological flow conditions enabled us to identify FimH residues crucial for adhesion. These results provide novel insights into screening methods to determine the effect of FimH mutants and potentially FimH antagonists.

**Electronic supplementary material:**

The online version of this article (doi:10.1007/s10096-016-2820-8) contains supplementary material, which is available to authorized users.

## Introduction

Infection with *Escherichia coli* is the most frequent cause of septicaemia in humans and commonly originates from the urinary tract [1]. Uropathogenic *E. coli* (UPEC) adhere to bladder epithelium in a process mediated by type 1 fimbriae via FimH engaging uroplakin 1a on urothelium, leading to urinary tract infection [2, 3]. Subsequently, FimH promotes invasion and is critical for blood-borne dissemination to other tissues [4]. Thus, in neonatal meningitis, FimH is essential for the localisation of UPEC to brain microvascular endothelium and invasion of the meninges [5, 6]. This establishes the pathogenic importance of FimH-mediated adhesion beyond the urinary tract.

FimH is located at the tip of type 1 fimbriae expressed by Gram-negative pathogens, including *E. coli*, *Salmonella enterica* and *Klebsiella pneumoniae* [7, 8]. It has two domains: an *N*-terminal lectin domain of FimH containing the mannose binding pocket (MBP) responsible for bacterial adhesion to cellular ligands and a C-terminal pilin domain that connects FimH to the fimbrial rod [9]. Introduction of shear stress after initial binding induces allosteric interactions between the lectin and pilin domains that increase the affinity of mannose for the MBP through a catch bond mechanism [10, 11].

Over the past decade, mutated FimH have been used extensively to probe the molecular basis for its binding to mannose, most commonly in studies performed under static conditions using yeast agglutination [12], FimH binding to pure mannose substrates [7, 10] or bacterial adhesion to bladder carcinoma cell lines [13] as end points. These have provided considerable insights into the molecular basis for the MBP binding with mannose, but necessarily poorly reflect physiological conditions in which it normally takes place. Specifically, there is a lack of data on FimH-dependent bacterial adhesion to microvascular endothelium that is thought to underlie blood-borne dissemination of *E. coli* [4]. We addressed this issue by generating and validating a panel of multiply disabled *E. coli* strains that uniquely express type 1 fimbriae and normal or mutated FimH [14], and systematically analysing the ability of the mutant strains to adhere to microvascular endothelium and bladder epithelium, under both static conditions and physiological shear stress. We show that FimH-dependent adhesion to endothelium occurs much more efficiently than to bladder epithelium and identify MBP residues that are critical for adhesion under shear stress but without detectable effects in static assays. Our results characterise important differential effects of FimH-mediated adhesion to different cellular substrates that reflect the different physiological conditions they are exposed to in vivo.

## Materials and methods

### Chemicals

Alpha-D-mannopyranoside (mannoside), RNase B (tri-mannosylated-3 M) and bovine serum albumin (BSA) were from Sigma-Aldrich (St. Louis, MO, USA). D-Mannose-BSA (mono-mannose-1 M) (14 atom spacer) was from Dextra Laboratories (Reading, UK), and 0.05 % Trypsin-EDTA and HEPES were from Life Technologies (Carlsbad, CA, USA).

### Antibodies

The following polyclonal antibodies were used for Western blot: uroplakin 1a (ABIN955479, 1:100, antibodies-online, Atlanta, GA, USA) and beta-actin (A2066, 1:500, Sigma-Aldrich). Secondary antibodies conjugated with alkaline phosphatase were from Promega (1:5000, Madison, WI, USA).

### Cell lines

Human dermal microvascular endothelial cells (G1S1) and conditionally immortalised glomerular endothelial cells (GEnC) were cultured using standard validated methods [15, 16]. GEnC were propagated at 33 °C (proliferation phase) and differentiated at 37 °C for 5 days prior to each experiment [15]. The human transitional cell carcinoma cell lines 5637 (ATCC HTB-9) and HT-1376 [17] and SV40-transformed urothelial cell line SV-HUC [18] were all kind gifts from Michael Wirth (University of Vienna).

### Bacterial strains and GFP labelling of bacteria

*Escherichia coli* were labelled with green fluorescence protein (GFP) using phage 1 transduction of gfp:bla from *E. coli* strain OS56 into the multiply disabled *E. coli* MS528 (*E. coli* MG1655 Δfim Δflu) [19], resulting in strain *E. coli* MSC95. The gfp gene was inserted into the chromosome of MS528 using the Lambda Red System with the lambda red proteins encoded on the plasmid pTP223, which includes a gene for tetracycline resistance [20, 21] (kindly provided by Antony Poteete). As the source of the drug resistance cassette, pKD4 carrying a kanamycin cassette was used [22].

*Escherichia coli* MSC95 completely devoid of all fimbriae was used as the FimH-negative control strain. *Escherichia coli* MSC95 expressing FimH (MSC95-FimH) was derived from *E. coli* PC31 *fimH* [23] located on the pMAS4 plasmid, together with pPKL115 carrying the entire fim gene cluster with a knock-out mutation in *fimH* [24] and used as standard FimH-bearing control strain.

### Site-directed mutagenesis of *fimH*

Mutations were introduced into the *fimH* gene from *E. coli* PC31 carried by the pMAS4 plasmid [23] by site-directed mutagenesis using the Phusion Site-Directed Mutagenesis Kit (F-541, Thermo Scientific, Waltham, MA, USA), following the manufacturer’s instructions. Specific primers were designed for each desired point mutation. For the expression of type 1 fimbriae carrying the mutated FimH protein, plasmids carrying the mutated *fimH* gene were individually transformed into MSC95 containing the plasmid pPKL115 carrying the *fim* gene cluster with a knock-out mutation in *fimH* [24]. Recombinant strains were grown in LB medium supplemented with 10 μg/ml chloramphenicol and 100 μg/ml ampicillin.

### Yeast agglutination assay

The ability of the recombinant *fimH* mutant strains to express a D-mannose binding phenotype was examined by yeast agglutination using an established method [25]. Briefly, 20 μl of 1 % (v/w) yeast in PBS were mixed with 20 μl of serial dilutions (non-diluted up to 1:16) of bacterial suspensions in PBS (normalised to OD_600_ = 0.3) of either MSC95-FimH or mutant strains on a microscopy slide, and the dilution at which agglutination occurred was recorded.

### Bacterial adhesion under static conditions

GFP-expressing *E. coli* MSC95-FimH (4 × 10^6^ CFU) were incubated with confluent mammalian cell lines in 12-well cell culture plates for 30 min on ice to prevent internalisation. The cultures were then washed three times with PBS to remove non-adherent bacteria before the mammalian cells were detached with trypsin-EDTA and the resulting single-cell suspensions were analysed by flow cytometry (LSRFortessa SORP, Becton Dickinson, San José, CA, USA). Bacterial adherence was quantified from the intensity of the GFP signal from single endothelial or urothelial cells identified by forward/sideward scatter, thus excluding GFP signals associated with cell clusters and/or from free bacteria. The results were analysed with FlowJo (Tree Star, Inc., Ashland, OR, USA) and expressed as the adhesion index, defined as the percentage of GFP-positive *E. coli* bound to mammalian cells. The adhesion of mutant *E. coli* strains to the cell lines was expressed in the results as a percentage of adhesion of the MSC95-FimH parent strain. The mannose dependence of adhesion was assessed by suspending the *E. coli* in media containing 4 % mannoside for 10 min on ice before the experiment.

### Bacterial adhesion under flow conditions

Vena8 Fluoro+ biochips (Cellix, Dublin, Ireland) were coated overnight with either 200 μg/ml D-mannose-BSA (1 M), 100 μg/ml RNAse B with high 3-mannose (3 M) residues or 2 % BSA alone at 4 °C and blocked prior to use with PBS + 0.2 % BSA. A total of 1 × 10^6^ *E. coli* prepared as described above were added to the substrates. The biochips were set up and washed according to the manufacturer’s instructions using the VenaFlux Assay Software (Cellix). The specificity of binding was assessed by pre-incubating *E. coli* MSC95-FimH with 2 % mannoside in PBS for 10 min prior to the experiment. Adhesion of bacteria under a shear stress of 1 dyne/cm^2^ was recorded every second in phase contrast and the settings were equal for both 1 M and 3 M (exposure time 344 ms, magnification 20×) for 5 min using an Axiovert 200M microscope (Zeiss, Oberkochen, Germany) with AxioVision 4.5 software. The total number of adherent bacteria per high-power field (HPF) was counted manually using ImageJ [26].

To assess FimH-dependent adhesion to mammalian cells under flow conditions, Vena8 Endothelial 8-channel biochips (Cellix), 800 nm long and 120 nm wide, were sterilised by UV-light and coated with FNC coating buffer (AthenaES, Baltimore, MD, USA) at 4 °C overnight. Cells were seeded into the biochips at 5 × 10^5^ cells per channel and allowed to adhere for 1 h, resulting in confluent cell layers. The cells were incubated for another 24 h in the biochip connected to the Kima pump (Cellix) with the following shear stress conditions: for bladder epithelial cells, 150 μl/min for 6 min, followed by 20 min of absence of flow; for microvascular endothelial cells, 450 μl/min for 6 min, followed by 20 min of absence of flow. Both were incubated at 37 °C with 5 % CO_2_. Bacterial samples were prepared as described for the 1 M and 3 M assays. The flow chamber was then connected to the Mirus Evo Nanopump (Cellix) and the channels were rinsed three times with 25 μl of media prior to each experiment, and bacterial adhesion was initiated by the addition of 1 × 10^6^ of bacterial suspension. As above, adhesion of bacteria was recorded every second under a shear stress of 1 dyne/cm^2^ in phase contrast and the settings were equal in all conditions (exposure time 344 ms, magnification 32×) for 5 min. In some experiments (stop/flow), 1 dyne/cm^2^ was exerted and paused for 5 min once bacteria were observed in the HPF, before 1 dyne/cm^2^ shear stress was re-applied. This, however, induced some gaps between the cells; any *E. coli* adhering to this were excluded, as mentioned above. The total numbers of *E. coli* that were adherent were counted as above. *Escherichia coli* were considered adherent when they were adhering for at least five frames at the end of the 5 min. Excluding criteria were *E. coli* that adhered to any plastic surface.

### Transmission electron microscopy

Transmission electron microscopy (TEM) was performed to confirm the expression of intact fimbriae on all mutant strains. Five independent TEM micrographs (final magnification 60,000×) were analysed from each mutant strain and were used to count the number of fimbriae along three circumferential areas of 500 nm each in which individual fimbriae were clearly distinguishable. The total number of fimbriae was then calculated from the circumferential outline of the bacteria (∼4500 nm) using ImageJ.

### Structural modelling

The crystal structure of FimH (PDB ID: 2VCO) [27] was uploaded in Visual Molecular Dynamics [28] (VMD, University of Illinois, Urbana–Champaign, IL, USA). The residues that we experimentally tested were highlighted.

### Statistical analyses

All calculations were made using GraphPad Prism 5.0 (GraphPad Prism Software, La Jolla, CA, USA) and *p*-values <0.05 were considered significant. Absolute values for the number of adherent bacteria were summarised as means ± standard error of the mean (SEM) and the significance of differences between them was assessed by Student’s *t*-test. Adhesion index assays were expressed as medians with interquartile ranges and illustrated using box and whisker plots. The overall significance was tested by Kruskal–Wallis followed by, when appropriate, individual two-tailed Mann–Whitney tests. In both cases, individual pairwise *p*-values were corrected for multiple comparisons using the Benjamini–Hochberg method, with a false discovery rate of α set to 0.05 [29]. For the correlation data, the average adhesion of the mutant strains was analysed; MSC95-FimH and MSC95 were excluded from the correlation calculations.

## Results

### Generation and characterisation of *E. coli* strains expressing mutated FimH

Using MSC95-FimH as the parent strain [23], we generated a panel of 14 GFP-tagged *E. coli* strains that uniquely express type 1 fimbriae with different alanine point mutations in the MBP of FimH (listed in Table 1). The residues that were selected for mutation directly interact with mannose (N46, D47, P49, D54, Q133, N135 and D140) and have been shown to bind to bladder tissue [27, 30, 31]. Two other residues (E50 and T53) were characterised in their ability to agglutinate yeast [32] and were included to examine their influence on MBP–mannose interactions. Three further residues are located at the back of the MBP, but their influence on adhesion was only analysed regarding mannose binding (Y55 and T57 [32]) or not at all (V56). The use of this panel of FimH mutations allowed us to systemically analyse the influence of each residue on adhesion to mammalian cells and binding to mannosylated substrates, and relate our results to previous findings.

**Table 1:**
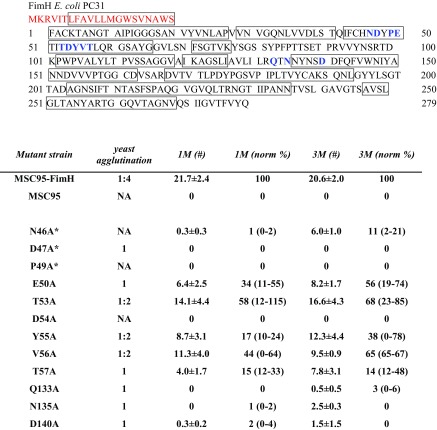
Overview of the mutations generated in MSC95-FimH and adhesion to 1 M and 3 M

The ability of *E. coli* FimH mutants to adhere to mannose substrates under flow and to agglutinate yeast. Binding properties were comparable in all three assays; however, some mutants exhibited marked differences in their ability to bind either 1 M or 3 M. Titres for yeast agglutination was the highest dilution at which agglutination still occurred. The adhesion of mutant *E. coli* to 1 M and 3 M was assessed, and the raw numbers of binding *E. coli* were normalised (norm) against *E. coli* numbers obtained in the same experiment with MSC95-FimH. Data represent mean % (range: lower limit %–upper limit %,). MBP: Mannose binding pocket; 1 M: D-mannose-BSA; 3 M: RNase containing 3-mannose residues; NA: no agglutination. An asterisk (*) indicates that the strains had dysmorphic fimbriae

**Fig. 1:**
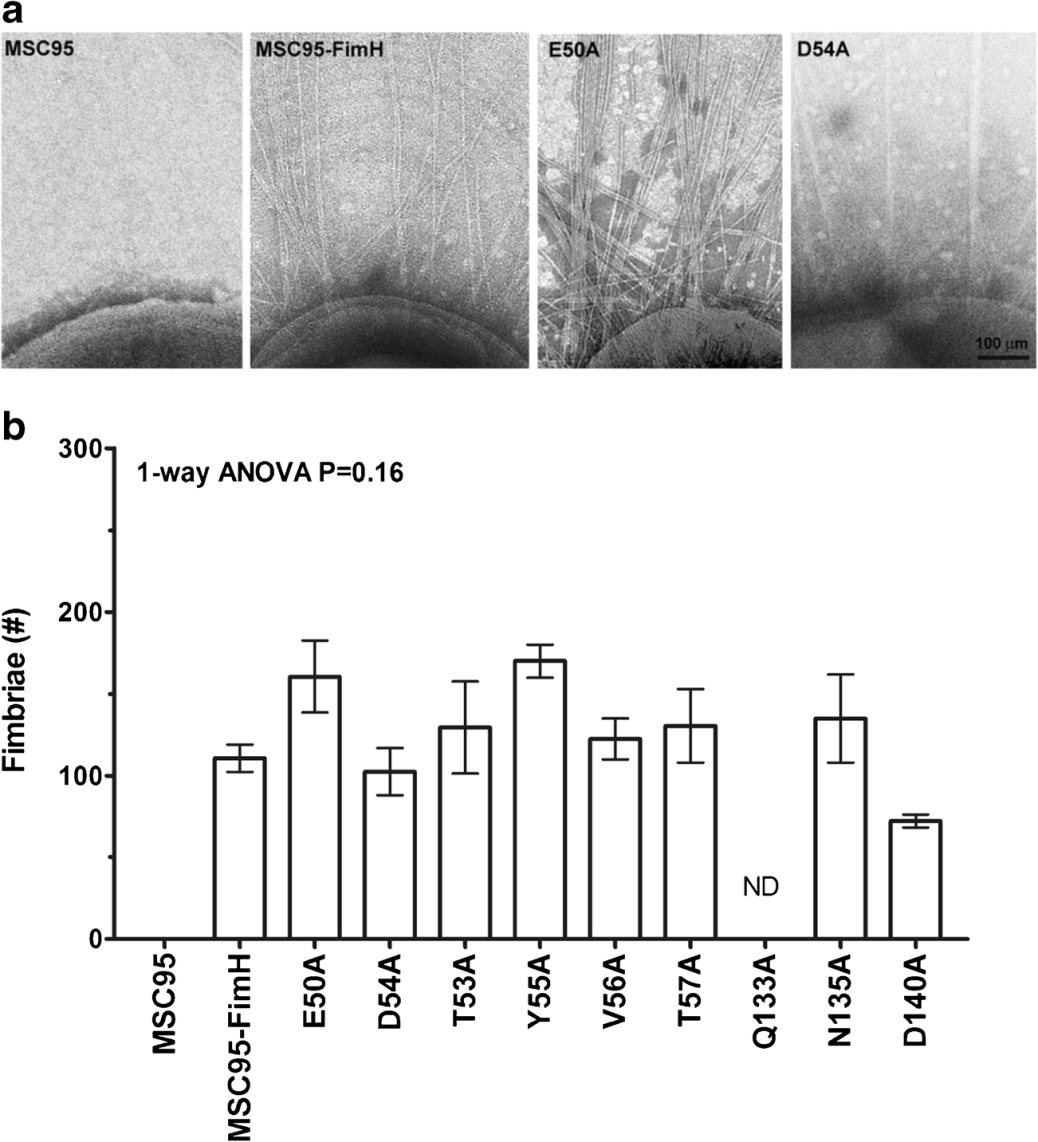
Characterisation of FimH mutant strains. **a** Representative images of the different mutants expressing type 1 fimbriae. **b** The number of fimbriae around the circumference of the parent strain MSC95-FimH was 110.7 ± 22.1. None of the numbers of fimbriae in the remaining mutant strains differed significantly from the parent strain (one-way ANOVA *p* = 0.16, drawn from the same population). The graph is representative of three independent experiments. The fimbriae of five different bacteria were counted per strain. Data are shown as mean ± standard error of the mean (SEM). *ND* not done

Since FimH mutations can disrupt fimbriogenesis [33], we assessed the integrity of the fimbriae of the mutant FimH strains by electron microscopy. Three strains had reduced numbers of dysmorphic fimbriae (N46A, D47A and P49A) and were excluded from further analysis. The remaining nine mutant strains had quantitatively and qualitatively normal fimbriae when compared to the parental MSC95-FimH strain (one-way ANOVA *p* = 0.24) (Fig. 1) and were used to analyse bacterial adhesion to yeast, to biochemical substrates and to mammalian cells.

### FimH-dependent bacterial adhesion to 1 M and 3 M

First, we tested FimH function by the established yeast agglutination assay, which provides a swift but semi-quantitative assessment [12, 34]. While D54A did not bind yeast, weak agglutination was observed for E50A, T57A, Q133A and N135A. The mutants T53A, Y55A and V56A adhered yeast with moderate titres, when compared to the native FimH bearing strain MSC95-FimH (Table 1). MSC95 lacking fimbriae did not cause yeast agglutination. The mutants analysed here have different yeast agglutination patterns, but do not necessarily reflect in vivo conditions [35].

**Fig. 2:**
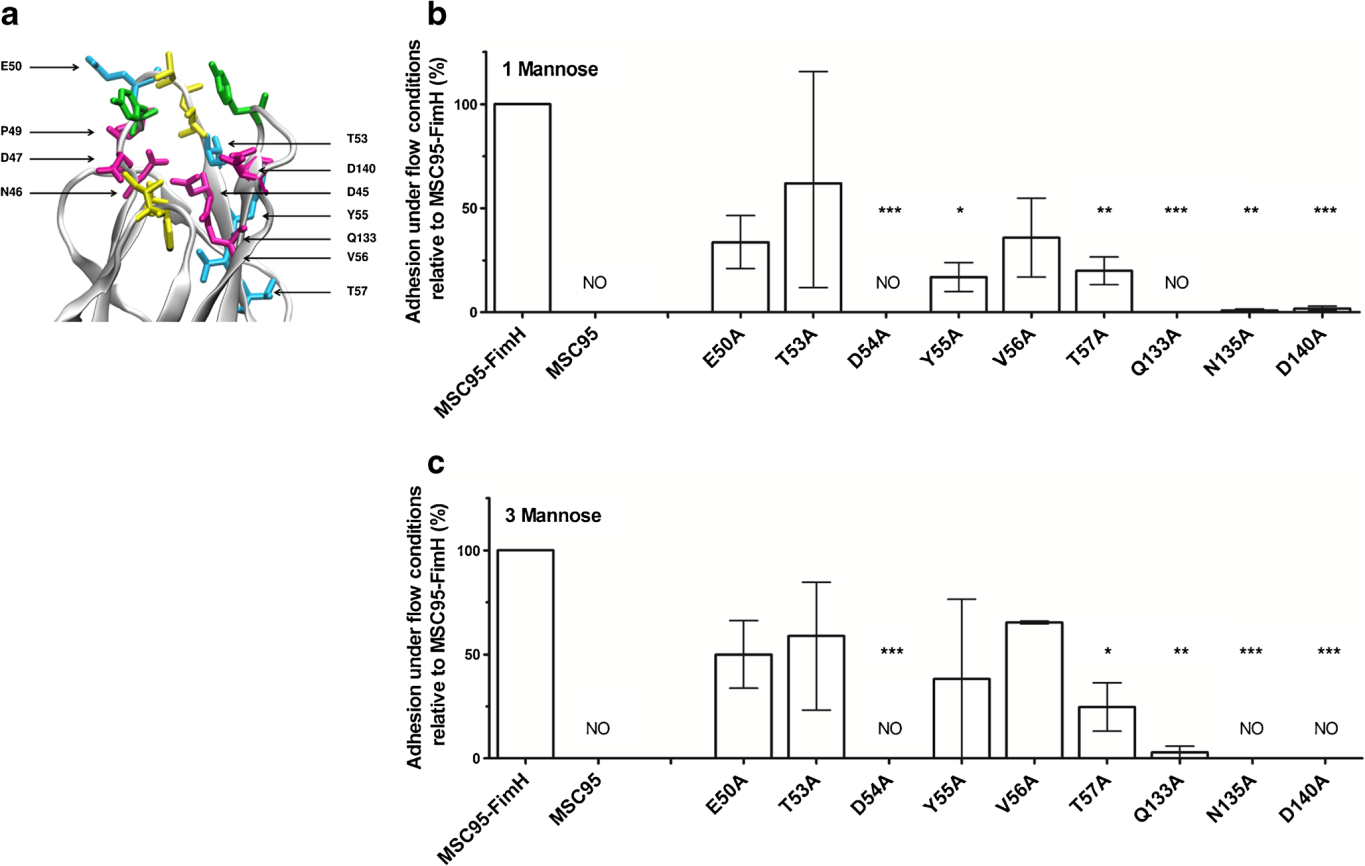
Mutations in FimH alter adhesion to 1 M-BSA and 3 M (RNAse B) under shear stress conditions. **a** The location of the mutations in this study are depicted in this crystal structure of lectin domain FimH (PDB entry: 2VCO). The different mutants were in the mannose binding pocket (*purple*) and in the flanking region (*teal*). Other functional FimH regions are tyrosine gate (*green*) and the hydrophobic ridge (*yellow*). **b**, **c** The binding of MSC95-FimH mutant strains to D-mannose-BSA (1 M, *white bars*) and RNase B containing 3-mannose residues (3 M, *black bars*) was determined under shear stress conditions. Adhesion of bacteria under a shear stress of 1 dyne/cm^2^ was recorded every second in phase contrast for 5 min with equal settings for both 1 M and 3 M, and the total number of adherent bacteria per high power field (HPF) was counted. Mutations in the mannose binding pocket of FimH resulted in profound differences of adhesion when compared to MSC95-FimH. The *bars* represent the mean percentage of adhesion of the different mutations compared to MSC95-FimH (100 %) ± range (min–max). Each strain was analysed at least three times in triplicate. *NO* no binding observed

Next, we quantified FimH-mediated adherence to mannosylated substrates under flow conditions [10] that should better reflect physiological conditions in vivo. Binding to mono-mannosylated (1 M) proteins is crucial for uropathogenic *E. coli*, while binding to tri-mannosylated (3 M) proteins has been reported to be important for non-uropathogenic *E. coli* for colonisation elsewhere [36, 37]. The MSC95-FimH parental strain showed strong adherence to yeast and efficiently bound to the 1 M substrate, D-mannose-BSA (21.7 ± 2.4 bacteria/HPF), and to the 3 M substrate, RNase B (20.6 ± 2.0 bacteria/HPF). The MBP mutations showed abrogated adhesion to 1 M (range 0–58 %) and 3 M (range 0–68 %) compared to the parent strain MSC95-FimH (Fig. 2). The results of two tests correlated in five of the nine strains tested: E50A and T53A bound moderately to yeast and to both 1 M [34 % (11–55 %) and 58 % (12–115 %) of MSC95-FimH binding, respectively] and 3 M [56 % (19–74 %) and 68 % (23–85 %)] (Table 1). In contrast, D54A did not agglutinate yeast nor did it bind to 1 M or 3 M. Mutant D140 was able to weakly agglutinate yeast but did not bind to 1 M [2 % (0–4 %)] or 3 M (0 %). Mutant Y55A agglutinated yeast but was severely impaired in binding to both 1 M [17 % (10–24 %)] and 3 M [38 % (0–78 %)] (Table 1). V56A, Q133A and N135A weakly agglutinated yeast but showed either moderate binding to 1 M [V56A; 44 % (0–64 %)] or none [Q133A; 0 % and N135A; 1 % (0–2 %)], with similar binding to 3 M [65 % (65–67 %), 3 % (0–6 %) and 0 %, respectively]. Mutant strains with dysmorphic fimbriae did not bind to 1 M or 3 M and did not agglutinate yeast (N46A, P49A) or only weakly (D47A) (Table 1).

### FimH-dependent bacterial adhesion to cells under static conditions

**Fig. 3:**
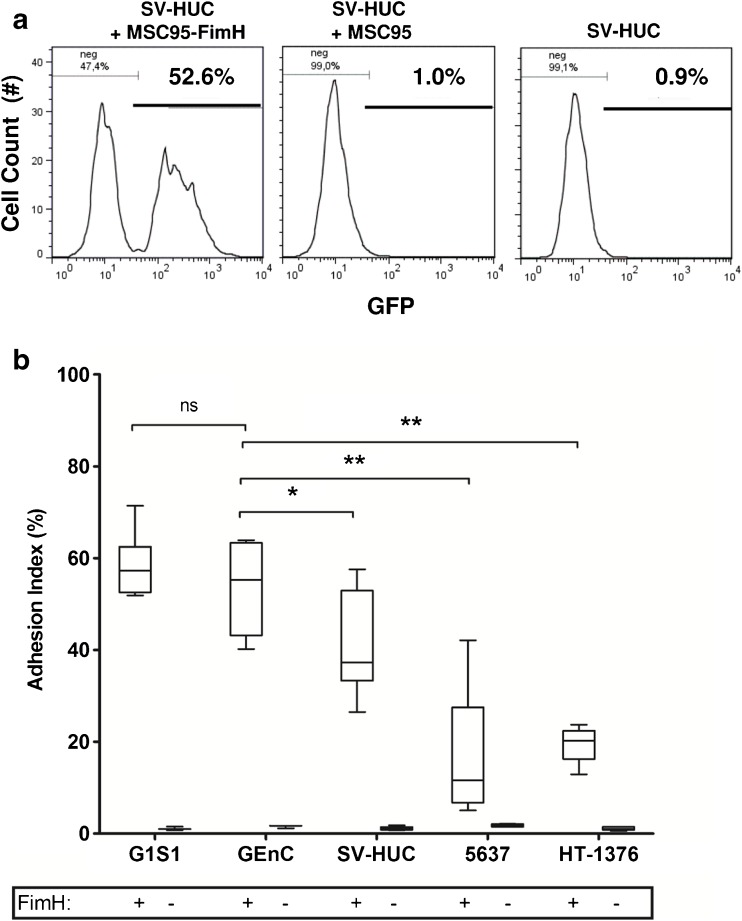
Adhesion of MSC95-FimH to cells under static conditions. **a** FACS analysis demonstrates that GFP-labelled FimH-positive *E. coli* (MSC95-FimH) adhere to the normal bladder epithelial cell line SV-HUC under static conditions (appearance of GFP positive peak, *left panel*), while FimH-negative *E. coli* (MSC95) (absence of GFP positive peak, *middle panel*) do not. *Right panel* SV-HUC alone. **b** MSC95-FimH (*white boxes*, FimH+) adhere better to human dermal microvascular (G1S1) and immortalised glomerular endothelial cells (GEnC) as analysed by FACS than to SV-HUC, and only poorly to malignant urothelial cells (5637 and HT-1376). MSC95 (FimH-negative) confirms that adherence is FimH-dependent. *p < 0.05, **p < 0.01, Kruskal–Wallis test followed by two-tailed Mann–Whitney test. Adhesion was determined to each cell line in at least four experiments in triplicate

We analysed the effect of different FimH mutations on adhesion to the relevant cell types by adapting the standard flow cytometry-based bacterial adhesion assay performed under static conditions [38]. FimH-dependent adhesion of GFP-tagged *E. coli* was quantified using an adhesion index calculated from the percentage of mammalian cells with adherent bacteria (Fig. 3a). Multiply-disabled MSC95-FimH adhered significantly better to endothelium derived from skin (G1S1) or glomeruli (GEnC) [G1S1; adhesion index 57.3 %, interquartile range (IQR) 51.9–71.4; GEnC; 55.2 % (IQR 40.2–63.9)] than to bladder urothelial cell lines, derived from normal bladder (SV-HUC; 37.3 %; IQR 26.5–57.6, *p* < 0.001 to G1S1, *p* < 0.01 to GEnC) or bladder carcinomas (5637 cells; 11.6 %; IQR 6.7–27.5, *p* < 0.01 to GEnC and HT-1376; 20.2 %; IQR 16.3–22.4, *p* < 0.01 to GEnC). Adhesion was both FimH- and mannose-dependent because it did not occur in the absence of FimH expression and was abrogated by pre-incubation with 4 % mannoside (Fig. 3b). Despite the strong expression of uroplakin 1a, the primary receptor for FimH on urothelium (Fig. S1), adhesion to urothelial cells was significantly less than to endothelial cells. To determine if the differences remained under physiological conditions, we analysed the adhesion of MSC95-FimH to urothelial and endothelial cells under flow.

### FimH-dependent bacterial adhesion under shear stress conditions

**Fig. 4:**
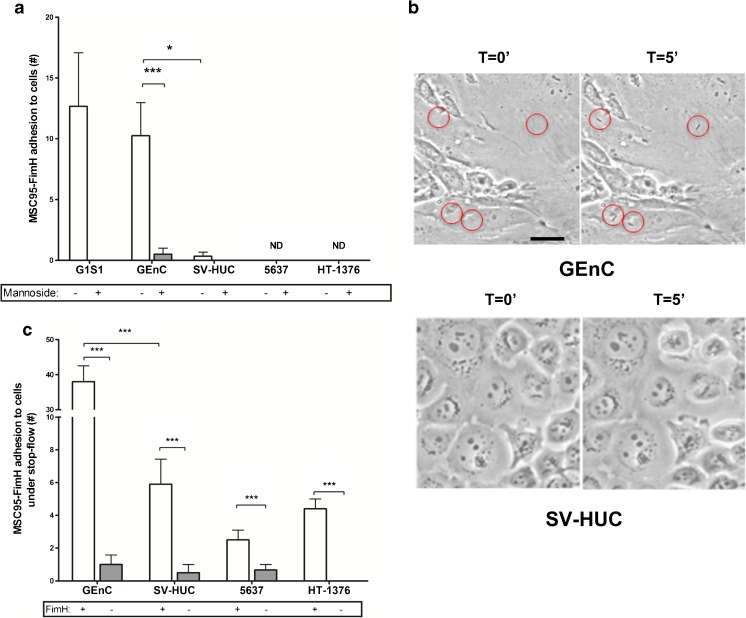
Adhesion of MSC95-FimH to cells under shear stress conditions. **a** MSC95-FimH (*white bars*) adhere to endothelial but not urothelial cells under shear stress conditions. More *E. coli* adhere to glomerular endothelial cells (GEnC) when compared to urothelial cells (SV-HUC). Data are shown as mean ± SEM. *ND* = no binding detected. *p < 0.05, ***p < 0.001. **b** Adhesion of bacteria under a shear stress of 1 dyne/cm^2^ was recorded every second in phase contrast for 5 min with equal settings for GEnC (*top*) and SV-HUC (*bottom*), and the total number of adherent bacteria per high power field (HPF) was counted. Adhesion to each cell line was determined in at least four experiments in triplicate. **c** Five minutes of static conditions followed by flow (stop/flow assay) allowed MSC95-FimH (*white bars*) to adhere to benign (SV-HUC) and malignant urothelial cells (5637, HT-1376). Data are shown as mean ± SEM. ***p < 0.001. Adhesion is mannose-dependent both under shear stress (**a**) and depends on FimH presence in stop/flow conditions (**b**)

Blood-borne bacteria encounter endothelium under shear stress, estimated to be around 1 dyne/cm^2^ in both glomerular and dermal capillaries [39, 40]. At this rate of flow, MSC95-FimH adhered effectively to confluent monolayers of G1S1 and GEnC in microfluidic flow chambers (G1S1: 12.6 ± 4.4 bacteria/HPF, GEnC: 10.2 ± 2.7) (Fig. 4a, b). Adhesion occurred rapidly without the rolling behaviour that is characteristic of leukocyte adhesion and, once adherent, the bacteria did not detach. Again, adhesion was entirely FimH- and mannose-dependent (data not shown and Fig. 4a). Unexpectedly, MSC95-FimH did not adhere to any of the bladder cell lines under shear stress (Fig. 4a, b). To stimulate the conditions of bladder voiding, we allowed MSC95-FimH to adhere for 5 min under static conditions before applying flow (stop/flow assay). MSC95-FimH adhered effectively under static conditions and was not dislodged from either bladder or endothelial cells by subsequent flow conditions, up to 15 dyne/cm^2^ (data not shown), but MSC95-FimH still adhered more efficiently to GEnC in this assay (GEnC: 38.0 bacteria/HPF; SV-HUC: 5.9; 5637 cells: 2.5; and HT-1376 cells: 4.4) (Fig. 4c).

### Adhesion of mutant FimH to mammalian cells under static and shear stress conditions

**Fig. 5:**
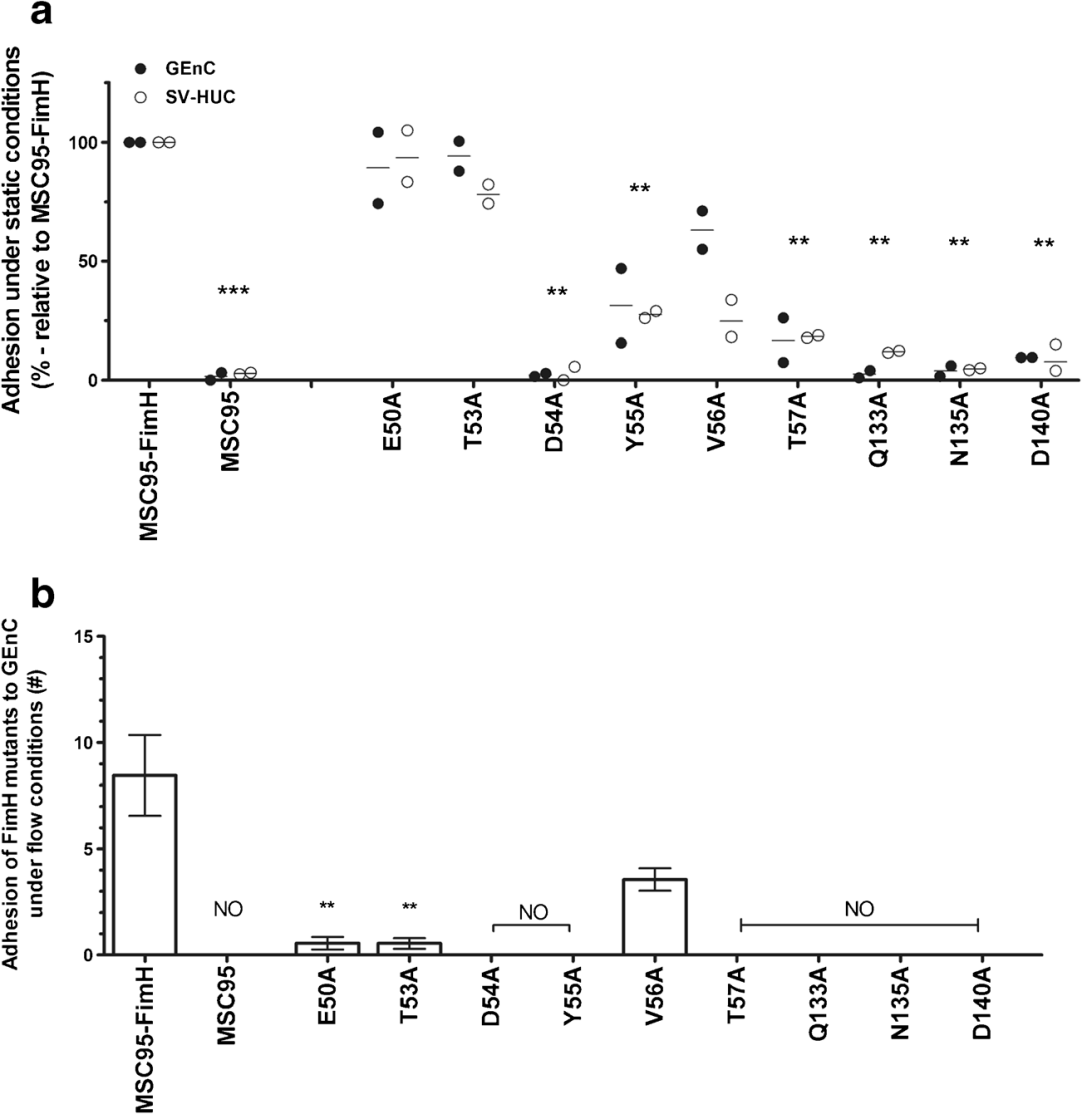
FimH mutations influence adhesion to endothelial and urothelial cells under static and shear stress conditions. **a** Adhesion of MSC95-FimH and nine FimH strains with mutations in the MBP was analysed under static conditions followed by FACS to glomerular endothelial (GEnC, *white bars*) and urothelial cells (SV-HUC, *black bars*). Mutations show abrogated binding, similar in both endothelial and urothelial cells. Data are shown as the average measurements of two individual experiments done in triplicate with the mean. *p < 0.05, **p < 0.01 compared to MSC95-FimH (Kruskal–Wallis followed by Mann–Whitney tests). Each strain was analysed two times in triplicate and individual *p*-values were corrected by the Benjamini method. **b** The amount of MSC95-FimH and FimH mutant *E. coli* strains that adhered to glomerular endothelial cells (GEnC) per HPF was counted after 5 min of shear stress. Data are shown as mean ± SEM. *p < 0.05, **p < 0.01 compared to MSC95-FimH (Student’s *t*-test). *NO* no binding observed. Each strain was analysed three times in triplicate

We then measured the ability of the FimH mutant strains to adhere to SV-HUC and GEnC in our static adhesion assay. Seven of the strains (MSC95, D54A, Y55A, T57A, Q133A, N135A and D140A) adhered less well, between 0 and 31.3 % of MSC95-FimH, to GEnC and SV-HUC (Fig. 5a). By contrast, alanine substitution of E50 and T53 had little or no effect (Fig. 5a) and adhesion to both cell lines was inhibited by mannoside, excluding the acquisition of novel mannose-independent binding sites as an explanation (Fig. S2). The remaining mutant strain V56A showed partial [63 % (55–71 %)] adhesion compared to the MSC95-FimH to GEnC, but this was also not due to mannose-independent binding (Fig. 5a and S2).

The effect of MBP mutations was even more pronounced under shear stress. Alanine substitution abrogated adhesion to GEnC in all mutant strains (Fig. 5b). Remarkably, the E50A and T53A mutants adhered normally under static conditions but had over 90 % reduced adhesion under shear stress (0.6 ± 0.3 and 0.5 ± 0.2 bacteria/HPF, respectively) compared to 8.5 ± 1.9 of MSC95-FimH (both *p* < 0.001, Student’s *t*-test). Only V56A that retained 63 % of its adhesive properties to GEnC under static conditions also adhered to these cells under flow [3.6 ± 0.5 bacteria/HPF (*p* = 0.08)] or 42 % of MSC95-FimH (Fig. 5b).

MSC95-FimH did not adhere to SV-HUC under shear stress and, so, we used the stop/flow assay to compare the adhesion of the mutants. Again, most strains did not adhere in this condition (Fig. S3). E50A and T53A mutants that adhered normally under static conditions exhibited around 50 % reduced adhesion (3.5 ± 1.5 and 6.0 ± 1.0 bacteria/HPF, respectively) compared to 8.8 ± 1.1 of MSC95-FimH. The only mutant strain not affected was V56A, which adhered more effectively than the parent strain (14.0 ± 1.0).

### Correlation between FimH mutant strains analysed with different methods

The correlation of the FimH mutants (Fig. S4) between adhesion to 1 M and the static adhesion to cells was 0.96 for GEnC and 0.75 for SV-HUC (Spearman, *p* = 0.0002 and *p* = 0.026, respectively). Between static adhesion and to 3 M, the correlation was similar; 0.84 for GEnC and 0.64 for SV-HUC (*p* = 0.006 and *p* = 0.066, respectively). The correlation of the mutant strains between adhering under static or shear stress conditions to GEnC was 0.59 (*p* = 0.097). The correlation for the different mutants to GEnC under flow was equal for both 1 M and 3 M; 0.71 for 1 M and 0.72 for 3 M (both *p* = 0.037). The adhesion of the mutants to SV-HUC in the stop/flow assay correlated well with adhesion to GEnC under continuous flow; 0.76 (*p* = 0.021) but less with adhesion under static conditions to SV-HUC; 0.55 (*p* = 0.133). Finally, the correlation between adhesion to 1 M and 3 M was 0.90 (*p* = 0.0020). Thus, there is a strong correlation between the adhesion on mannosylated substrates and static adhesion to cells. However, the more stringent conditions of adhesion under flow revealed stronger effects of FimH mutations that were masked when adhesion was analysed under static conditions. The results demonstrate the critical influence of MBP residues on bacterial adhesion to endothelium under shear stress conditions, even if there is a high correlation between SV-HUC and GEnC; 0.91 (*p* = 0.0013) (Fig. S4). They also show that binding to mannosylated substrates is not a true reflection of the effect of FimH mutations. Therefore, an assay in which cells are used mimicking the physiological conditions encountered in vivo is more informative and provides additional insight into the adhesive properties of FimH variants.

## Discussion

Type 1 fimbriated pathogens, like *E. coli*, adhere to a variety of biological surfaces, both epithelia and endothelia. Accordingly, they have adapted by modifying the sequence and structure of adhesins like FimH to enable improved adhesion under different—static or shear stress—conditions [10, 41]. Our study explores the methodologies used to analyse FimH-mediated adhesion of *E. coli* using mutations in the MBP of FimH [31]. We first used yeast agglutination as an established screening assay [34] to test the functionality of mutant FimH and found that, to a varying degree, all our mutant strains were able to adhere to yeast. These results were comparable to those obtained with the more physiological binding to mannosylated substrates and mammalian cells under both static and shear stress conditions, which confirmed that three mutant strains lacking intact fimbriae were unable to adhere. All our strains were engineered to bear type 1 fimbriae only and the differences observed in these assays cannot be attributed to other pili, whose possible influence remains enigmatic. They confirm, however, the necessity for additional investigations to establish the adhesive properties of (mutant) FimH. The analysis of FimH-mediated adhesion under flow to coated substrates [42, 43] or cells [44] was established previously. In this study, by exposing FimH-expressing *E. coli* under shear stress conditions to endothelial cells seeded onto biochips, we identified potential novel effects of FimH mutations.

We established two assays that enabled us to compare adhesion to pure mannosylated substrates and relevant urothelial and endothelial cells under static and shear stress conditions: in the first assay, similar to a recently described method [38], we allowed FimH-bearing *E. coli* to adhere to mammalian cells under static conditions and analysed the numbers of bacteria attached by FACS. The second assay measured the adherence under shear stress by allowing bacteria to bind 1 M or 3 M substrates and cells under flow. We found that the results between static assays that measure adhesion to mannosylated substrates or mammalian cells correlated well. But there were distinct differences when we compared the results of wild-type or mutated FimH-bearing *E. coli* adhering to cells under static conditions to those under shear stress conditions.

FimH mediates the adhesion of *E. coli* to brain endothelial cells and is a virulence factor critical for blood-borne dissemination of *E. coli* [4]. We show that FimH also mediates efficient bacterial adhesion to human microvascular endothelium of the skin and the glomerulus of the kidney under static conditions and during physiological shear stress. In agreement with other studies, bacteria adhered better under conditions of high shear stress (1 dyne/cm^2^), which reflects the physiological condition [40], than low shear stress (0.2 dyne/cm^2^) [7, 44]. Once attached, adherent bacteria could not be dislodged. It is known that shear stress enhances the strength of FimH-mediated adhesion through allosteric coupling [41, 45, 46], which could contribute to the failure of shear stress to dislodge adherent MSC95-FimH. The high affinity binding of terminal mannose residues on cellular receptors to MBP of FimH is the central event in *E. coli* adhesion.

Our panel of *E. coli* MSC95-FimH strains with unique FimH mutations enabled us to identify, in addition to those described [10], amino acids that differentially modulated the effect of shear stress. The ability of FimH mutations to disrupt fimbriogenesis is a potential limitation to this approach [33] that affected three of our strains. We measured the agglutination potential of these mutant strains and found that they correlated relatively well with other reports [31]. Minor differences could lie in the glycosylation of guinea pig red blood cells compared to yeast, as reported by others [47–49]. Regardless, the results with the remaining nine strains with normal fimbriae demonstrated that the mannose binding pocket is crucial for the adhesion of *E. coli* to endothelial cells [31]. Comparison of adhesion to endothelium under static and shear stress conditions revealed that E50 and T53 were essential under shear stress. This only partly correlated with binding to mannosylated substrates under identical assay conditions, demonstrating that there are important differences for the adhesion of isolated mannosylated substrates and mammalian cells. E50 has been demonstrated to not interact with the sugar ligand, but to stabilise R98, which is, thereby, oriented to interact with the ligand [50]. Due to its location on the backbone of the MBP, T53 interactions with 3-mannose are blocked by the side chains of I52 and D54. The effect of T53 mutations can, therefore, only be indirect through changes in its backbone energetic minimum, which could lead to gentle changes in the 3-mannose binding site [27, 50]. This supports a model in which these amino acid residues contribute to initial interaction between FimH and its cellular receptors, and that facilitates subsequent insertion of their terminal mannose residues into the MBP to establish firm adhesion. This would be consistent with the lack of reported mutations of these residues in clinical *E. coli* isolates, suggesting an important evolutionary conserved contribution to virulence [51].

The *E. coli* strain MSC95-FimH adhered efficiently to microvascular endothelium both under static conditions and shear stress, whereas adhesion to all three urothelial cell lines tested in static conditions was less efficient and appeared abrogated by flow. The observation that MSC95-FimH fails to adhere to bladder epithelial cells under flow is in apparent contrast to an earlier study [44]. However, the discrepancy probably relates to technical differences between the two studies and specifically to our use of an *E. coli* strain specifically engineered to express type 1 fimbriae exclusively, which enabled us to examine the unique effects of FimH.

In our experiments, differences between FimH-mediated adhesion to urothelial cells under static and shear stress conditions could be regarded, as the experimental results correlate stop/flow conditions that occur in the urinary bladder during voiding. Allowing MSC95-FimH to adhere under static conditions before applying shear stress resulted in an increase of bacteria bound to the non-neoplastic urothelial cell line SV-HUC and, to a lesser extent, to the neoplastic cell lines 5637 and HT1376 that were not dislodged by flow. Using this setup, we confirmed the reduced adhesion capabilities of E50A and T53A, although the effect was less obvious than during continuous flow. This supports the model in which these residues contribute to mannose binding. When mutated, FimH is limited in making contact with mannose residues under shear stress conditions.

In summary, we show that, by analysing bacterial adhesion under physiologically relevant conditions, novel effects of FimH mutations are observed. Our study thus highlights the fact that the results obtained from investigating FimH adhesion are dependent on the methodological differences of the assays used and the physiological situation they represent. The results with MSC95-FimH, expressing native or mutated variants of FimH, identified the importance of the MBP that critically influences the adhesion under flow, and provide novel insights into screening methods to determine the effect of FimH mutants and potentially FimH antagonists.

## Electronic supplementary material

Below are the links to the electronic supplementary material.

Fig. S1 (GIF 6 kb)

**High-resolution image (TIF 1358 kb)**

Fig. S2 (GIF 35 kb)

**High-resolution image (TIF 4728 kb)**

Fig. S3 (GIF 31 kb)

**High-resolution image (TIF 4454 kb)**

Fig. S4 (GIF 97 kb)

**High-resolution image (TIF 11786 kb)**

ESM 5 (DOCX 12 kb)

## References

[1] Farrell DJ, Morrissey I, De Rubeis D, Robbins M, Felmingham D (2003). A UK multicentre study of the antimicrobial susceptibility of bacterial pathogens causing urinary tract infection. J Infect.

[2] Xie B, Zhou G, Chan SY, Shapiro E, Kong XP, Wu XR, Sun TT, Costello CE (2006). Distinct glycan structures of uroplakins Ia and Ib: structural basis for the selective binding of FimH adhesin to uroplakin Ia. J Biol Chem.

[3] Wu XR, Sun TT, Medina JJ (1996). In vitro binding of type 1-fimbriated Escherichia coli to uroplakins Ia and Ib: relation to urinary tract infections. Proc Natl Acad Sci U S A.

[4] Smith SN, Hagan EC, Lane MC, Mobley HL (2010) Dissemination and systemic colonization of uropathogenic Escherichia coli in a murine model of bacteremia. MBio 1(5). pii: e00262-10. doi:10.1128/mBio.00262-10 10.1128/mBio.00262-10 PMC2993011 21116344

[5] Khan NA, Kim Y, Shin S, Kim KS (2007). FimH-mediated Escherichia coli K1 invasion of human brain microvascular endothelial cells. Cell Microbiol.

[6] Johnson JR, Russo TA (2002). Uropathogenic Escherichia coli as agents of diverse non-urinary tract extraintestinal infections. J Infect Dis.

[7] Stahlhut SG, Tchesnokova V, Struve C, Weissman SJ, Chattopadhyay S, Yakovenko O, Aprikian P, Sokurenko EV, Krogfelt KA (2009). Comparative structure–function analysis of mannose-specific FimH adhesins from Klebsiella pneumoniae and Escherichia coli. J Bacteriol.

[8] Kisiela D, Laskowska A, Sapeta A, Kuczkowski M, Wieliczko A, Ugorski M (2006). Functional characterization of the FimH adhesin from Salmonella enterica serovar Enteritidis. Microbiology.

[9] Knight SD, Bouckaert J (2009). Structure, function, and assembly of type 1 fimbriae. Top Curr Chem.

[10] Nilsson LM, Thomas WE, Trintchina E, Vogel V, Sokurenko EV (2006). Catch bond-mediated adhesion without a shear threshold: trimannose versus monomannose interactions with the FimH adhesin of Escherichia coli. J Biol Chem.

[11] Yakovenko O, Sharma S, Forero M, Tchesnokova V, Aprikian P, Kidd B, Mach A, Vogel V, Sokurenko E, Thomas WE (2008). FimH forms catch bonds that are enhanced by mechanical force due to allosteric regulation. J Biol Chem.

[12] Schembri MA, Sokurenko EV, Klemm P (2000). Functional flexibility of the FimH adhesin: insights from a random mutant library. Infect Immun.

[13] Scharenberg M, Abgottspon D, Cicek E, Jiang X, Schwardt O, Rabbani S, Ernst B (2011). A flow cytometry-based assay for screening FimH antagonists. Assay Drug Dev Technol.

[14] Klemm P, Christiansen G (1987). Three fim genes required for the regulation of length and mediation of adhesion of Escherichia coli type 1 fimbriae. Mol Gen Genet.

[15] Satchell SC, Tasman CH, Singh A, Ni L, Geelen J, von Ruhland CJ, O’Hare MJ, Saleem MA, van den Heuvel LP, Mathieson PW (2006). Conditionally immortalized human glomerular endothelial cells expressing fenestrations in response to VEGF. Kidney Int.

[16] Schoppmann SF, Soleiman A, Kalt R, Okubo Y, Benisch C, Nagavarapu U, Herron GS, Geleff S (2004). Telomerase-immortalized lymphatic and blood vessel endothelial cells are functionally stable and retain their lineage specificity. Microcirculation.

[17] Rasheed S, Gardner MB, Rongey RW, Nelson-Rees WA, Arnstein P (1977). Human bladder carcinoma: characterization of two new tumor cell lines and search for tumor viruses. J Natl Cancer Inst.

[18] Christian BJ, Loretz LJ, Oberley TD, Reznikoff CA (1987). Characterization of human uroepithelial cells immortalized in vitro by simian virus 40. Cancer Res.

[19] Kjaergaard K, Schembri MA, Hasman H, Klemm P (2000). Antigen 43 from Escherichia coli induces inter- and intraspecies cell aggregation and changes in colony morphology of Pseudomonas fluorescens. J Bacteriol.

[20] Murphy KC (1998). Use of bacteriophage lambda recombination functions to promote gene replacement in Escherichia coli. J Bacteriol.

[21] Murphy KC, Campellone KG, Poteete AR (2000). PCR-mediated gene replacement in Escherichia coli. Gene.

[22] Datsenko KA, Wanner BL (2000). One-step inactivation of chromosomal genes in Escherichia coli K-12 using PCR products. Proc Natl Acad Sci U S A.

[23] Schembri MA, Pallesen L, Connell H, Hasty DL, Klemm P (1996). Linker insertion analysis of the FimH adhesin of type 1 fimbriae in an Escherichia coli fimH-null background. FEMS Microbiol Lett.

[24] Pallesen L, Poulsen LK, Christiansen G, Klemm P (1995). Chimeric FimH adhesin of type 1 fimbriae: a bacterial surface display system for heterologous sequences. Microbiology.

[25] Wu KH, Wang KC, Lee LW, Huang YN, Yeh KS (2012). A constitutively mannose-sensitive agglutinating Salmonella enterica subsp. enterica serovar typhimurium strain, carrying a transposon in the fimbrial usher gene stbC, exhibits multidrug resistance and flagellated phenotypes. Scientific World J.

[26] Schneider CA, Rasband WS, Eliceiri KW (2012). NIH Image to ImageJ: 25 years of image analysis. Nat Methods.

[27] Wellens A, Garofalo C, Nguyen H, Van Gerven N, Slättegård R, Hernalsteens JP, Wyns L, Oscarson S, De Greve H, Hultgren S, Bouckaert J (2008). Intervening with urinary tract infections using anti-adhesives based on the crystal structure of the FimH-oligomannose-3 complex. PLoS One.

[28] Humphrey W, Dalke A, Schulten K (1996). VMD: visual molecular dynamics. J Mol Graph.

[29] Benjamini Y, Hochberg Y (1995). Controlling the false discovery rate: a practical and powerful approach to multiple testing. J R Stat Soc Ser B Methodol.

[30] Bouckaert J, Berglund J, Schembri M, De Genst E, Cools L, Wuhrer M, Hung CS, Pinkner J, Slättegård R, Zavialov A, Choudhury D, Langermann S, Hultgren SJ, Wyns L, Klemm P, Oscarson S, Knight SD, De Greve H (2005). Receptor binding studies disclose a novel class of high-affinity inhibitors of the Escherichia coli FimH adhesin. Mol Microbiol.

[31] Hung CS, Bouckaert J, Hung D, Pinkner J, Widberg C, DeFusco A, Auguste CG, Strouse R, Langermann S, Waksman G, Hultgren SJ (2002). Structural basis of tropism of Escherichia coli to the bladder during urinary tract infection. Mol Microbiol.

[32] Kisiela DI, Rodriguez VB, Tchesnokova V, Avagyan H, Aprikian P, Liu Y, Wu XR, Thomas WE, Sokurenko EV (2013). Conformational inactivation induces immunogenicity of the receptor-binding pocket of a bacterial adhesin. Proc Natl Acad Sci U S A.

[33] Munera D, Palomino C, Fernández LA (2008). Specific residues in the N-terminal domain of FimH stimulate type 1 fimbriae assembly in Escherichia coli following the initial binding of the adhesin to FimD usher. Mol Microbiol.

[34] Yadav PR, Tyagi R (2005). Immuno-biotechnology of yeast cells.

[35] Chen SL, Hung CS, Pinkner JS, Walker JN, Cusumano CK, Li Z, Bouckaert J, Gordon JI, Hultgren SJ (2009). Positive selection identifies an in vivo role for FimH during urinary tract infection in addition to mannose binding. Proc Natl Acad Sci U S A.

[36] Sokurenko EV, Chesnokova V, Doyle RJ, Hasty DL (1997). Diversity of the Escherichia coli type 1 fimbrial lectin. Differential binding to mannosides and uroepithelial cells. J Biol Chem.

[37] Sokurenko EV, Chesnokova V, Dykhuizen DE, Ofek I, Wu XR, Krogfelt KA, Struve C, Schembri MA, Hasty DL (1998). Pathogenic adaptation of Escherichia coli by natural variation of the FimH adhesin. Proc Natl Acad Sci U S A.

[38] García Méndez KB, Bragagnolo G, O’Callaghan D, Lavigne JP, Keriel A (2016) A high-throughput assay for the measurement of uropathogenic Escherichia coli attachment to urinary bladder cells. Int J Exp Pathol 97(2):194–201. doi:10.1111/iep.12181 10.1111/iep.12181 PMC4926042 27273601

[39] Yao L, Salvucci O, Cardones AR, Hwang ST, Aoki Y, De La Luz Sierra M, Sajewicz A, Pittaluga S, Yarchoan R, Tosato G (2003). Selective expression of stromal-derived factor-1 in the capillary vascular endothelium plays a role in Kaposi sarcoma pathogenesis. Blood.

[40] Rops AL, Jacobs CW, Linssen PC, Boezeman JB, Lensen JF, Wijnhoven TJ, van den Heuvel LP, van Kuppevelt TH, van der Vlag J, Berden JH (2007). Heparan sulfate on activated glomerular endothelial cells and exogenous heparinoids influence the rolling and adhesion of leucocytes. Nephrol Dial Transplant.

[41] Thomas WE, Trintchina E, Forero M, Vogel V, Sokurenko EV (2002). Bacterial adhesion to target cells enhanced by shear force. Cell.

[42] Yakovenko O, Tchesnokova V, Sokurenko EV, Thomas WE (2015). Inactive conformation enhances binding function in physiological conditions. Proc Natl Acad Sci U S A.

[43] Thomas WE, Nilsson LM, Forero M, Sokurenko EV, Vogel V (2004). Shear-dependent ‘stick-and-roll’ adhesion of type 1 fimbriated Escherichia coli. Mol Microbiol.

[44] Aprikian P, Interlandi G, Kidd BA, Le Trong I, Tchesnokova V, Yakovenko O, Whitfield MJ, Bullitt E, Stenkamp RE, Thomas WE, Sokurenko EV (2011). The bacterial fimbrial tip acts as a mechanical force sensor. PLoS Biol.

[45] Le Trong I, Aprikian P, Kidd BA, Forero-Shelton M, Tchesnokova V, Rajagopal P, Rodriguez V, Interlandi G, Klevit R, Vogel V, Stenkamp RE, Sokurenko EV, Thomas WE (2010). Structural basis for mechanical force regulation of the adhesin FimH via finger trap-like beta sheet twisting. Cell.

[46] Rodriguez VB, Kidd BA, Interlandi G, Tchesnokova V, Sokurenko EV, Thomas WE (2013). Allosteric coupling in the bacterial adhesive protein FimH. J Biol Chem.

[47] Stahlhut SG, Struve C, Krogfelt KA, Reisner A (2012). Biofilm formation of Klebsiella pneumoniae on urethral catheters requires either type 1 or type 3 fimbriae. FEMS Immunol Med Microbiol.

[48] Ito T, Suzuki Y, Mitnaul L, Vines A, Kida H, Kawaoka Y (1997). Receptor specificity of influenza A viruses correlates with the agglutination of erythrocytes from different animal species. Virology.

[49] Lesage G, Bussey H (2006). Cell wall assembly in Saccharomyces cerevisiae. Microbiol Mol Biol Rev.

[50] Han Z, Pinkner JS, Ford B, Obermann R, Nolan W, Wildman SA, Hobbs D, Ellenberger T, Cusumano CK, Hultgren SJ, Janetka JW (2010). Structure-based drug design and optimization of mannoside bacterial FimH antagonists. J Med Chem.

[51] Schwartz DJ, Kalas V, Pinkner JS, Chen SL, Spaulding CN, Dodson KW, Hultgren SJ (2013). Positively selected FimH residues enhance virulence during urinary tract infection by altering FimH conformation. Proc Natl Acad Sci U S A.

