# Supplementary figures and images for: Adhesion of Escherichia coli under flow conditions reveals potential novel effects of FimH mutations
Source: Eur J Clin Microbiol Infect Dis. 2016 Nov 5;36(3):467–78. doi: 10.1007/s10096-016-2820-8 (PMC5309269; doi:10.1007/s10096-016-2820-8)

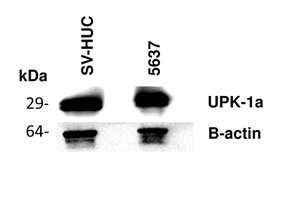

Supplement: Supplementary file 1 — (GIF 6 kb) [file 10096_2016_2820_Fig6_ESM.gif]

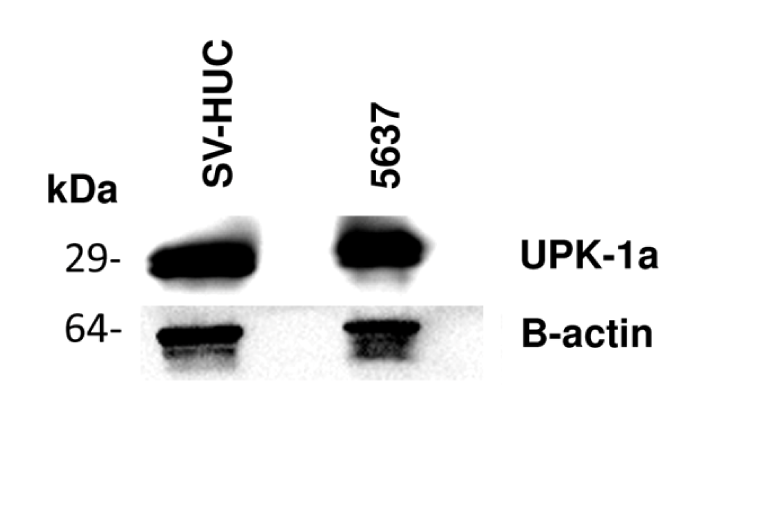

Supplement: Supplementary file 2 — High-resolution image (TIF 1358 kb) [file 10096_2016_2820_MOESM1_ESM.tif]

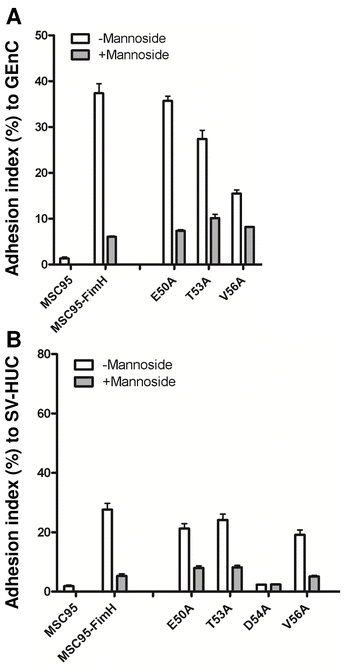

Supplement: Supplementary file 3 — (GIF 35 kb) [file 10096_2016_2820_Fig7_ESM.gif]

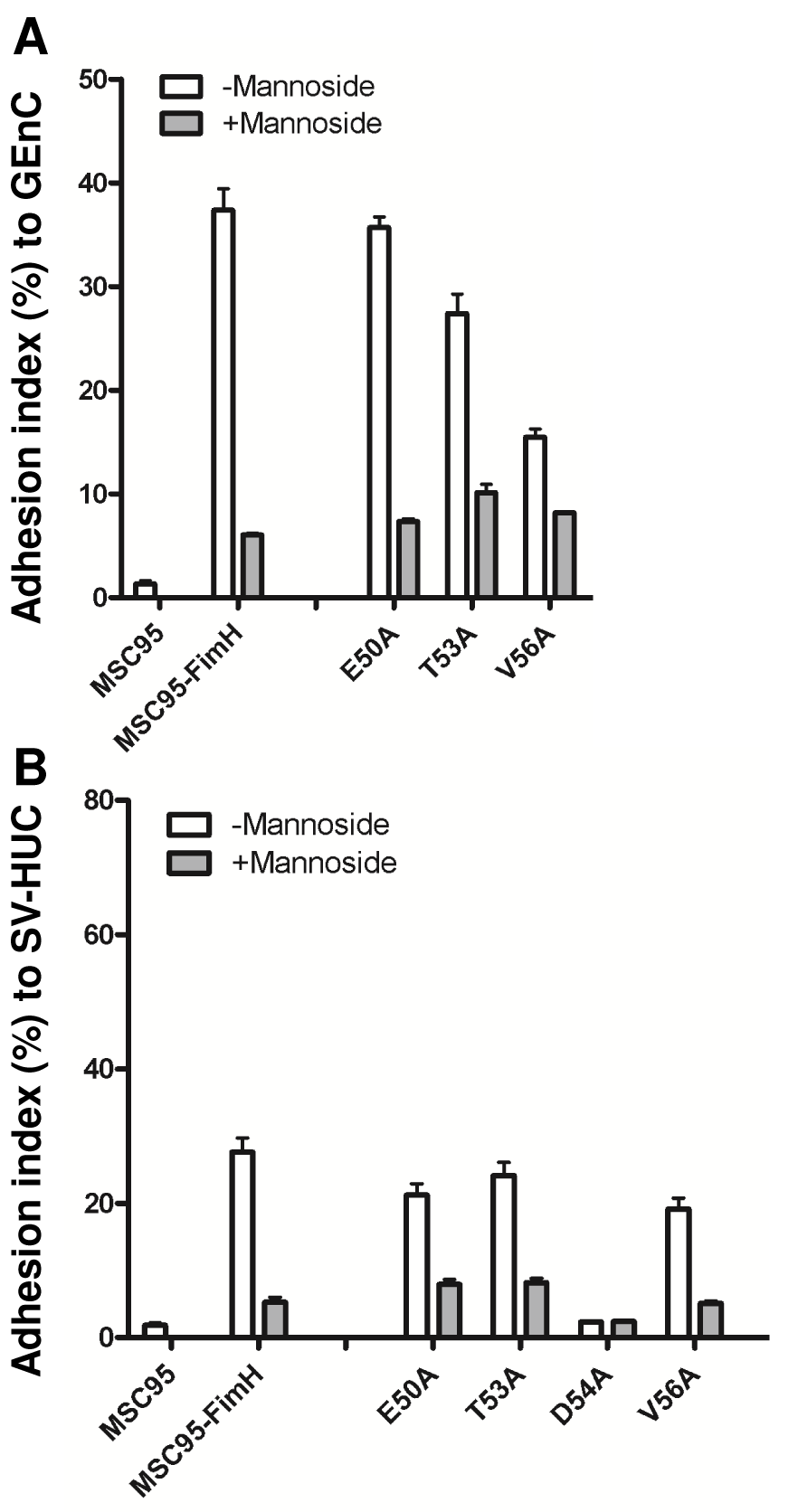

Supplement: Supplementary file 4 — High-resolution image (TIF 4728 kb) [file 10096_2016_2820_MOESM2_ESM.tif]

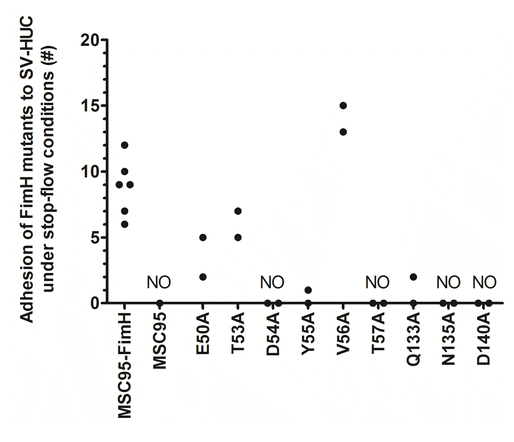

Supplement: Supplementary file 5 — (GIF 31 kb) [file 10096_2016_2820_Fig8_ESM.gif]

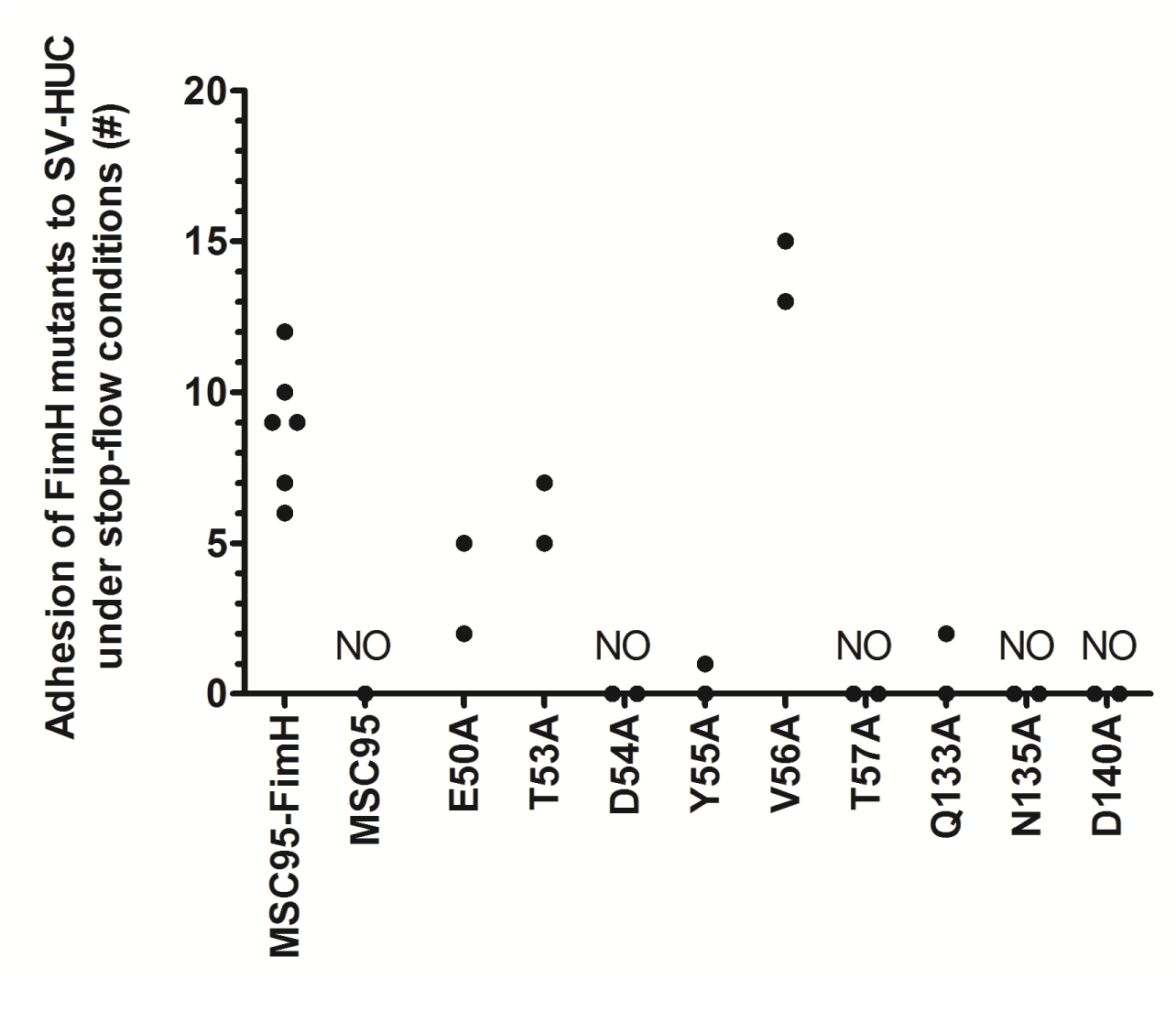

Supplement: Supplementary file 6 — High-resolution image (TIF 4454 kb) [file 10096_2016_2820_MOESM3_ESM.tif]

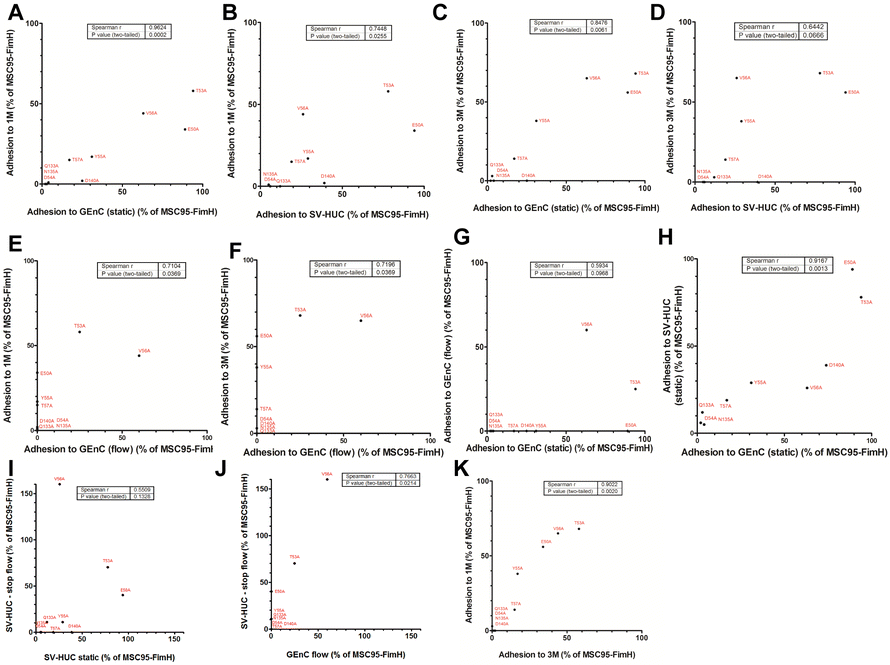

Supplement: Supplementary file 7 — (GIF 97 kb) [file 10096_2016_2820_Fig9_ESM.gif]

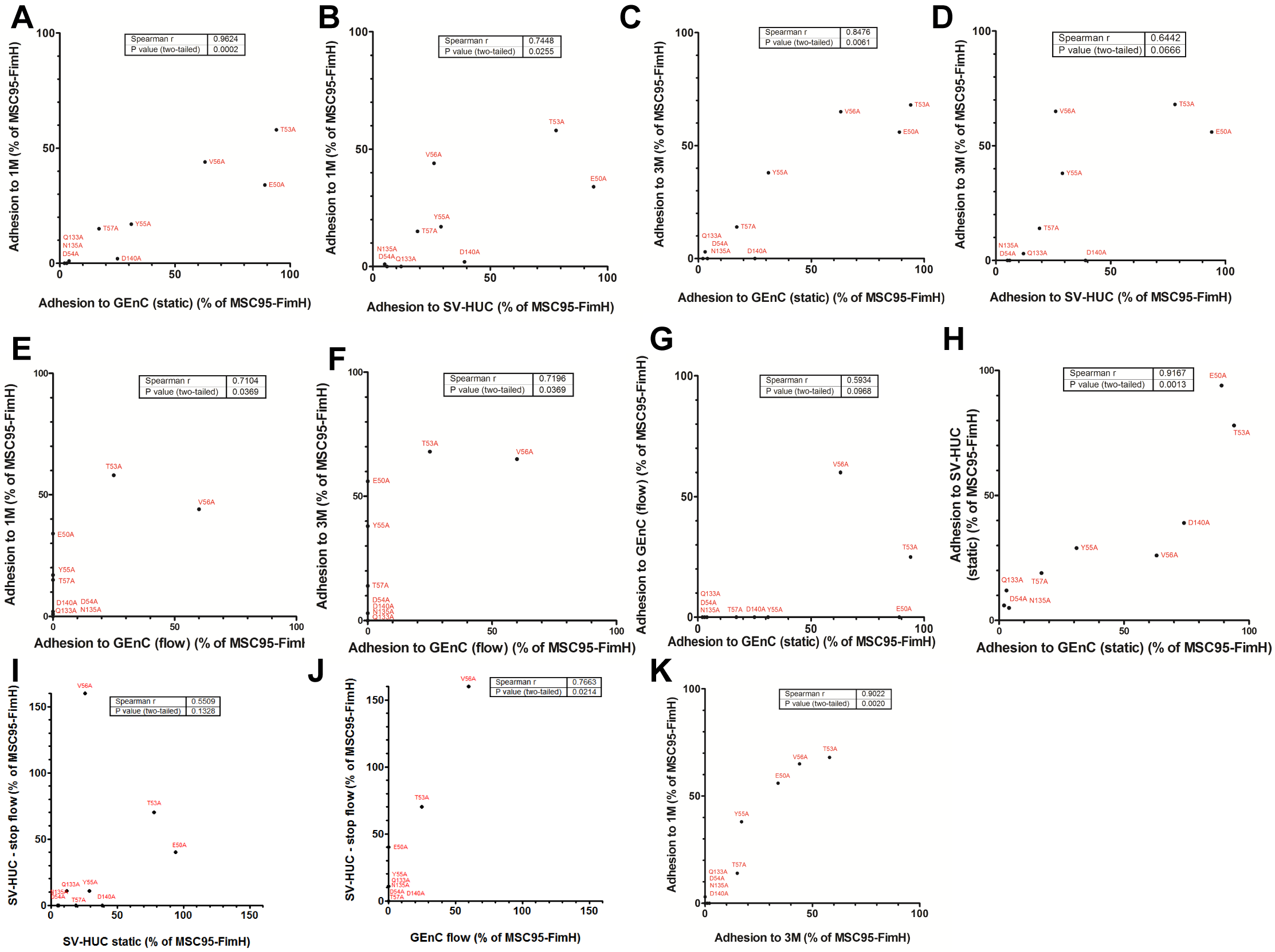

Supplement: Supplementary file 8 — High-resolution image (TIF 11786 kb) [file 10096_2016_2820_MOESM4_ESM.tif]
