# Supplementary material for: Adhesion of Escherichia coli under flow conditions reveals potential novel effects of FimH mutations
Source: Eur J Clin Microbiol Infect Dis. 2016 Nov 5;36(3):467–78. doi: 10.1007/s10096-016-2820-8 (PMC5309269; doi:10.1007/s10096-016-2820-8)
Supplement: Supplementary file 9 — (DOCX 12 kb) [file 10096_2016_2820_MOESM5_ESM.docx]

**Figure S1. Urothelial cell lines express Uroplakin 1a.** Native lysates were prepared for Western blot from SV-HUC and 5637 for uroplakin 1a with Beta-actin as a loading control. The blots are representative for two independent experiments.

**Figure S2. MSC95-FimH mutants retain mannose specificity in the static assay.** The adhesion of GFP labeled MSC95-FimH *E. coli* and a panel of adherent MSC95-FimH mutant strains was analysed under static conditions to SV-HUC (A) and GENC (B) with (grey) or without (white) mannoside pre-incubation. Adhesion is displayed as adhesion index. Each mutant with inhibitor was analyzed two times in triplicate.

**Figure S3.** **Adhesion of the mutants to SV-HUC under stop-flow conditions.** The amount of MSC95-FimH and FimH mutant strain *E. coli* that adhered to urothelial cells (SV-HUC) per high powered field were counted after 5 minutes of static (stop) and 2 minutes of flow conditions. NO: no binding observed. Each dot represents the mean of a duplicate experiment**.**

**Figure S4. Correlation between adhesion of FimH mutants compared between methods.** (A) The correlation between GEnC (static) and 1M. (B) the correlation between SV-HUC (static) and 1M. (C) the correlation between GEnC static and 3M. (D) the correlation between SV-HUC (static) and 3M. (E) the correlation between GEnC (flow) and 1M. (F) the correlation between GEnC (flow) and 3M. (G) The correlation between adhesion to GEnC under static and flow conditions. (H) the correlation between GEnC and SV-HUC under static conditions. (I) The correlation between SV-HUC under stop-flow and SV-HUC under static conditions. (J) The correlation between SV-HUC under stop-flow and GEnC under flow conditions. (K) The correlation between 1M and 3M. Written in red are the different mutants. For the correlation data, the mean adhesion of the mutants relative of MSC95-FimH was used. MSC95-FimH and MSC95 was not taken into account as all the adhesion data are relative of 100% MSC95-FimH. MSC95 was used as a negative control of adhesion and therefore not included.
